# Neural Indicators of Fatigue in Chronic Diseases: A Systematic Review of MRI Studies

**DOI:** 10.3390/diagnostics8030042

**Published:** 2018-06-21

**Authors:** María Goñi, Neil Basu, Alison D. Murray, Gordon D. Waiter

**Affiliations:** 1 Aberdeen Biomedical Imaging Centre (ABIC), Lilian Sutton Building, Foresterhill, University of Aberdeen, Aberdeen AB25 2ZN, UK; a.d.murray@abdn.ac.uk (A.M.); g.waiter@abdn.ac.uk (G.W.); 2 Health Science Building, Foresterhill, University of Aberdeen, Aberdeen AB25 2ZN, UK; neilbasu@abdn.ac.uk

**Keywords:** fatigue, magnetic resonance, chronic diseases

## Abstract

While fatigue is prevalent in chronic diseases, the neural mechanisms underlying this symptom remain unknown. Magnetic resonance imaging (MRI) has the potential to enable us to characterize this symptom. The aim of this review was to gather and appraise the current literature on MRI studies of fatigue in chronic diseases. We systematically searched the following databases: MedLine, PsycInfo, Embase and Scopus (inception to April 2016). We selected studies according to a predefined inclusion and exclusion criteria. We assessed the quality of the studies and conducted descriptive statistical analyses. We identified 26 studies of varying design and quality. Structural and functional MRI, alongside diffusion tensor imaging (DTI) and functional connectivity (FC) studies, identified significant brain indicators of fatigue. The most common regions were the frontal lobe, parietal lobe, limbic system and basal ganglia. Longitudinal studies offered more precise and reliable analysis. Brain structures found to be related to fatigue were highly heterogeneous, not only between diseases, but also for different studies of the same disease. Given the different designs, methodologies and variable results, we conclude that there are currently no well-defined brain indicators of fatigue in chronic diseases.

## 1. Introduction

The perception of fatigue is subjective, due to the interchangeable use of “tiredness” and the clinically pertinent experience of fatigue. There is no general consensus on the definition of fatigue. Nevertheless, for clinical purposes, it has been defined as an overwhelming feeling of physical and/or mental tiredness, along with a lack of energy which constrains the daily activities of the patient [1].

As opposed to weakness related to primary muscle disorders, fatigue has been conceived as a central nervous system event, considering that it remains after resting or sleeping. It may involve lack of attention, decline of executive and cognitive functions, difficulties in information processing or loss of productivity [2,3].

This symptom is often reported as one of the most burdensome, and the main cause of decrease in quality of life within chronic diseases, such as Rheumatoid Arthritis (RA) [4,5], Systemic Lupus Erythematosus (SLE) [6], primary biliary cirrhosis [7], or Human Immunodeficiency Virus (HIV) [8] among others.

There is a growing body of research that reports that aetiology of cognitive fatigue is multifactorial [9,10,11]. Whereas inflammation and disease activity have shown poor correlation with fatigue [12,13,14,15], variables such as pain [13,14,16,17,18,19,20,21], disability [12,16,17,21,22,23,24] and depression [13,14,25,26,27,28] among others appear to be commonly implicated in the complex process of this symptom. In summary, fatigue is a non-precise and greatly subjective symptom, difficult to specify, and therefore, arduous to measure and study.

Due to its multidimensional nature, a substantial number of questionnaires to assess fatigue have been developed [29,30]. These questionnaires may consist of a single-item measure, multi-item measures that assess further fatigue issues and calculate an overall score, or multidimensional measures with sub-scores for several domains of fatigue (e.g., mental, physical or emotional factors). Notwithstanding the broad number of fatigue questionnaires, these are subjective measurement tools. The absence of objective biological measures and the lack of understanding of the mechanisms underlying fatigue, are the greatest obstacles in the development of therapies.

Kluger et al. [31] proposed the concept of fatigability as an objective measure of fatigue, to be distinguished from subjective fatigue, assessed through self-rated measures. This objective fatigue may be, for example, the one related to neural activity. A way to assess fatigability is to subject the patient to sustained cognitive tasks [32,33].

Structural Magnetic Resonance Imaging (sMRI) is an accurate identifier of macroscopic diseases, but is limited in its ability to identify microscopic structural alterations in the brain, which are thought to be the most relevant to fatigue [34,35]. Nevertheless, other sophisticated MRI techniques can address this gap. In particular, Diffusion Tensor Imaging (DTI) has already reported some possible neural indicators of fatigue in chronic disorders such as Multiple Sclerosis (MS) [34] and Fibromyalgia (FM) [35] among others.

Another promising technique in the search for the brain structures involved in fatigue is functional Magnetic Resonance Imaging (fMRI). Here, the performance of cognitive tasks during fMRI has demonstrated several differences between fatigue and non-fatigue populations [36,37,38]. Furthermore, great promise in improving the usefulness of fMRI has been shown in the study of brain activity in the resting state [39,40].

Taken together, these MRI techniques provide a tool of inestimable value in the study of the brain mechanisms involved in fatigue, with its consequent potential as an objective biomarker. In spite of this fact, few studies have employed these techniques in the field of chronic diseases. Additionally, the diversity of study designs, interventions, techniques and analytical analysis makes it difficult to compare neural correlates of fatigue and hence, to confirm if the neurophysiology of fatigue is something generic or specific for each disease.

This systematic review (SR) aims to summarize those MRI studies applied in the search of neural indicators of fatigue in chronic diseases. Our main goals are: (1) to determine if there are well-defined neural indicators of fatigue in chronic diseases; (2) to determine if there are common fatigue indicators across chronic diseases; and given the variety of interventions and designs; (3) to determine which is the best approach to follow in the search of neural indicators of fatigue.

## 2. Methods

To ensure high quality reporting, the current systematic review adheres to the recommendation for systematic reviews of the PRISMA statement [41]. The literature search was based on a predefined list of search terms, inclusion, exclusion and quality criteria. The approaches used for data collection, extraction of characteristics and analysis of results are fully described.

### 2.1. Information Sources

A comprehensive search strategy was developed for studying fatigue within chronic diseases through neuroimaging techniques. Experimental and observational studies, with prospective or retrospective data collection, cross-sectional and longitudinal design, cohort-, case control- and randomized-designed studies were included in this review.

The review was carried out by searching electronic databases and through consultation with experts in the field and a medical librarian specialist. Studies were identified through Medline (Ovid Medline (R) 1946 to 22 April 2016), Embase (1974 to 27 April 2016), PsycInfo (1806 to 22 April 2016) and Scopus. All studies were included, without any language or date restriction. The last search was run on 28 April 2016.

### 2.2. Search

The following search terms were used for all databases: (fatigue OR lethargy OR weakness OR weariness OR debility OR enervation OR exhaustion OR faintness OR feebleness OR heaviness OR languor OR lassitude OR listlessness OR burnout OR fatigation OR tiredness OR overtired* OR asthenia) [title] AND (“magnetic resonance” OR MRI OR fMRI OR neuroimaging OR “diffusion tensor imaging” OR “voxel-based morphometry” OR “voxel based morphometry”) [title/abstract] AND (brain OR cerebr* OR neur*) [title/abstract] NOT (“multiple sclerosis” OR MS) [title].

### 2.3. Study Selection

In the search of neural changes related to fatigue, the SR was focused on the study of mental or cognitive fatigue, discarding physical fatigue due to muscular disorders. The non-invasive and non-ionizing nature, high resolution, great contrast between tissues and ability to detect subtle changes, makes the MRI one of the favourite tools in current clinical and research practice [42]. This SR focuses on MRI studies, excluding other neuroimaging techniques such as Computed Tomography (CT) or Positron Emission Tomography (PET). Another fact to take into account is that primary central nervous system (CNS) diseases generally induce gross structural abnormalities on MRI. Such abnormalities may mask some of the more subtle neural correlates (measured by more advanced MR protocols), which are expected to provide insight in the context of fatigue. Therefore, CNS diseases such as Parkinson’s disease, post-stroke syndrome, multiple sclerosis or amyotrophic lateral sclerosis among others were excluded.

As opposed to epidemiological studies, most of the neuroimaging studies make use of small sample sizes. However, good statistical power can still be achieved with sample sizes between 10–15 subjects [43,44]. In this review, studies with a minimum of 10 subjects were included.

Therefore, studies were included if they met the following criteria: (1) observational or interventional study design; (2) investigation of mental or cognitive fatigue; (3) application of structural or functional magnetic resonance imaging and (4) study of chronic disease. Studies were excluded if they: (1) study muscular or physical fatigue; (2) use non MR techniques (i.e., Positron Emission Tomography (PET), Computed Tomography (CT), etc.); (3) study primary CNS diseases (as Multiple Sclerosis (MS), Amyotrophic Lateral Sclerosis (ALS), stroke, etc.); (4) had sample sizes with less than 10 participants and (5) were single cases. After deletion of duplicates, the titles and abstracts of all records were reviewed.

### 2.4. Data Extraction

The specific outcomes extracted from each study included: (1) disease; (2) demographic characteristics of patients (number, male/female, age mean and standard deviation); (3) demographic characteristics of control group (number, male/female, age mean and standard deviation); (4) study design (cross-sectional or longitudinal); (5) period of follow-up (if applicable); (6) task during fMRI (if applicable); (7) method of assessment of fatigue; (8) imaging modality and (9) statistical approach.

### 2.5. Synthesis of Results

Brain correlates of fatigue were extracted for each of the studies. The outcomes were analysed according to: (1) illness and (2) neuroimaging techniques. Fatigue inducing tasks were reported for functional neuroimaging studies.

### 2.6. Quality Assessment

Those studies accomplishing the inclusion and exclusion criteria were evaluated for quality assessment using an established 10-item quality score [45]. The list of questions are: (1) Does the study have a clearly defined research objective? (2) Does the study adequately describe the inclusion criteria? (3) Does the study adequately describe the exclusion criteria? (4) Does the study report on the population parameters/demographics? (5) Does the study report details on assessment of fatigue? (6) Does the study provide details of imaging protocol? (7) Does the study provide a proper control group? (8) Does the study apply proper statistical analysis? Correction for multiple comparisons? (9) Does the study adequately report on the strength of the results? and (10) Do the authors report on the limitations of their study?

## 3. Results

### 3.1. Study Selection

The search of Medline, Embase, PsycInfo and Scopus databases provided a total of 1202 citations. After deleting duplicates, 727 papers remained. Of these, a total of 655 studies were discarded after reviewing titles and abstract, as they did not meet the inclusion or exclusion criteria. Two hundred and forty-seven were discarded for reporting single cases. Ninety-five of the studies were reviews, book chapters, did not have an abstract or were theoretical reports. A further 169 studies were excluded for being focused on muscular or physical fatigue. From the remaining papers, 38 more were discarded, as they studied fatigue in healthy participants only, and another 59 studies because they investigated non related illnesses (brain cancer, ALS, MS, infarction, stroke, clinically isolated syndrome, stress-related exhaustion, cocaine addiction or scleroderma). Twenty-two more studies were excluded for not studying fatigue, 7 for not using neuroimaging techniques, 17 more for using non related imaging techniques (PET, CT, etc.), and 1 because it focused on animal models. Finally, 72 remaining papers which met the inclusion and exclusion criteria were selected for full-text reading.

After reading the full papers, 46 studies were discarded. From them, 29 were meeting abstracts or posters or it was not possible to access the full text [46,47,48,49,50,51,52,53,54,55,56,57,58,59,60,61,62,63,64,65,66,67,68,69,70,71,72,73,74]. Another 6 of the papers used a very small number of patients [36,75,76,77,78,79], 6 of them did not fully define fatigue [80,81,82,83,84,85], 1 was a protocol [86], 1 a mini review [87], 2 of them employed non related imaging techniques [88,89] and another one studied physical fatigue [90].

**Figure 1:**
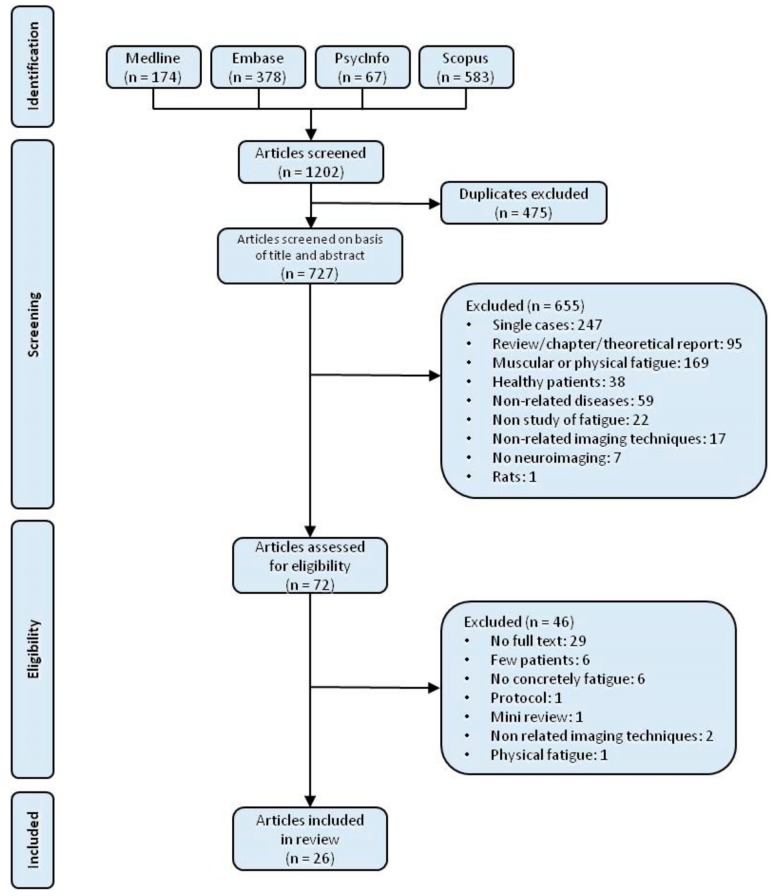
Flowchart of the review procedure.

Finally, 26 studies remained [39,40,91,92,93,94,95,96,97,98,99,100,101,102,103,104,105,106,107,108,109,110,111,112,113,114]. Figure 1 shows the process followed in this review.

### 3.2. Study Details and Characteristics

Table 1 summarizes the study characteristics pertinent to our research: demographic features, study design, duration of follow-up, task during the fMRI, fatigue assessment, imaging modality and statistic methods.

### 3.3. Quality Assessment

All 26 studies were assessed for quality (Table 2). Nineteen studies [39,40,91,93,95,96,97,98,100,101,103,106,107,108,110,111,112,113,114] were of high quality, as they met all the quality criteria. Four studies [99,102,104,109] were of very good quality and two of good quality [92,94]. One study [105] was judged to be of fair quality, because it had incomplete inclusion/exclusion criteria, lacked details of the imaging protocol, statistical assessment and did not have an analysis of the limitations of the study.

### 3.4. Synthesis of Results

Brain correlates of fatigue were extracted for each of the studies, and they are summarized in Table 3.

**Figure 2:**
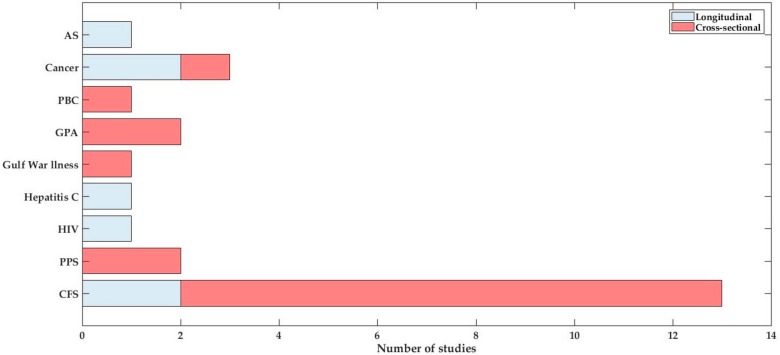
Distribution of longitudinal and cross-sectional studies per disease.

From a total of 26 studies, seven of the studies adopted a longitudinal approach, while the remaining ones were cross-sectional (Figure 2).

Next, the results are explained:

(a) Disease type

Neural correlates of fatigue in ankylosing spondylitis were mainly found in the parietal lobe, specifically in the inferior parietal sulcus [93] and postcentral gyrus [93,97], and the basal ganglia (caudate nucleus and putamen [97]). Other regions such as superior temporal polysensory area and insula were correlated with fatigue reduction after anti TNF-α therapy. Fatigue scores [97] were negatively correlated with GM volume in the dorsal and ventral attention networks, and positively correlated in the executive control network. In the case of granulomatosis with polyangiitis (GPA) [96], higher activation during a fatigue task in the medial frontal gyrus, paracentral lobule, globus pallidus and thalamus was found, while Basu et al. suggested fornix and cingulum play an important role in GPA-related fatigue [102]. With regard to chronic fatigue syndrome (CFS), results between studies were heterogeneous. Most of the significant correlates of fatigue were found in the frontal lobe (dorsolateral prefrontal cortex [94,110], dorsomedial prefrontal cortex [110], medial prefrontal cortex [111], lateral prefrontal cortex [91], medial superior frontal gyrus [106], and bilateral supplemental and premotor region [112]) and the limbic system (parahippocampal gyrus [39,110], anterior cingulate [39,40,106,111], posterior cingulate [39,40,110], and thalamus and hippocampus [39]). Other areas related to fatigue were found in the parietal lobe (supramarginal gyrus and postcentral gyrus [39], and superior parietal region [112]), midbrain [109] and globus pallidus [108]. Cope et al. found white matter lesions in a minority of all groups [114], while Zeineh et al. and De Lange et al. reported no significant findings [107,113]. Studies in other diseases reported varying results. Regarding cancer, dorsomedial prefrontal cortex [98] and executive network [99] were found to be related with fatigue. In [100] no significant findings were reported. Other correlates with cognitive fatigue were found in globus pallidus in the case of cirrhosis [101], ventral striatum in Hepatitis C [104] and inferior fronto-occipital fasciculus in Gulf War Illness [103], whereas in postpoliomyelitis (PPS), putamen, reticular formation and medial leminiscus was related with fatigue [92], while Trojan et al. did not find significant results [95]. Finally, basal ganglia was related to fatigue in HIV patients [105], although the quality of this study was judged to be only fair.

(b) Neuroimaging technique

FMRI was employed in nine of the studies: cancer [98,99], GPA [96] and CFS [106,108,110,111,112,113]. DTI was carried out in 5 of the studies: AS [97], cancer [98], GPA [102], Gulf War Illness [103] and CFS [107]. Functional connectivity was assessed in CFS [39,40,106]. MRS was employed in cancer [98,100], cirrhosis [101] and HIV [105]. Other techniques such as qMT were used in Hepatitis C [104] and MTR in cirrhosis [101]. The different fatigue-induced approaches during the fMRI included the Tower of London task [98], Paired Associates Memory Task [98], verbal working memory task [99], Paced Auditory Serial Attention Test (PASAT) [96,112], gambling [108], n-Back task [111] and a motor and visual imagery task [113].

## 4. Discussion

### 4.1. Summary of Evidence

In the present article, we have systematically reviewed the available MRI studies in chronic diseases where fatigue is a common burden. In the case of ankylosing spondylitis (AS), a form of arthritis, fatigue is considered as a significant symptom [21,115]. This symptom is so prevalent in AS that it has been suggested to be considered as an independent domain from other symptoms related to the disease, such as pain or impairment [116]. This review compiled two AS studies reporting fatigue-related structures [93,97]. Some of these structures, such as insula, dorsal and ventral attention network and the executive control network are related to cognitive functions, while other structures such as the primary sensory cortex, inferior parietal sulcus and superior temporal polysensory area are involved in sensory experience. Cancer-related fatigue is the most prevalent and debilitating symptom reported by patients [117]. Approximately 90% of patients treated with radiation and 80% of patients treated with chemotherapy suffer from fatigue [118]. In this review, we gathered three studies regarding cancer-related fatigue, although just two of them, relating to breast cancer, stated significant findings. Blesch et al. reported that 99% of breast cancer patients experienced some level of fatigue [119]. Dorsomedial prefrontal cortex and the executive network were found to be related to fatigue, being both structures involved in cognitive functions. With regard to primary biliary cirrhosis, a long-term liver disease, long term fatigue affects approximately 68–85% of the patients [7,120], considered as the worst or one of the worst symptoms in around half of them [120]. Only one paper studying fatigue correlates in PBC accomplished the inclusion criteria of this review. This paper reported the globus pallidus structure, associated with the regulation of voluntary movement, to be related with fatigue. Granulomatosis with Poliangiitis (GPA) is a rare multisystem autoimmune disorder of undetermined etiology. Although fatigue prevalence in GPA has not been reported, studies within other rheumatic disorders exhibited rates between 60% and 90% [121,122,123]. In this review, two studies regarding the GPA-related fatigue were presented. Basu et al. reported several brain regions related to fatigue, such as thalamus, paracentral lobule and the medial global pallidus [96]. These areas are mainly linked to sensory and motor coordination. Same authors found the fornix and cingulum, both part of the limbic system, involved in the experience of this symptom [102]. Gulf War Illness is a chronic and multisymptomatic illness that affects military veterans of the Gulf War. This disorder is characterized by a wide range of symptoms, including significant fatigue, muscle pain and cognitive issues among others [124]. The experience of permanent fatigue is frequently described by Gulf War veterans [125,126,127,128]. Kelsall et al. reported that up to 66% of Gulf War patients suffer from mild to severe fatigue [125]. Furthermore, between 1.6 and 5.1% of these patients met the chronic fatigue syndrome criteria [126,129,130]. This review found only one study related to fatigue in Gulf War Illness [103]. The study found a correlation between fatigue and axial diffusivity in the inferior fronto-occipital fasciculus. This brain structure is related to the integration of auditory and visual association. Hepatitis C is caused by the hepatitis virus which, in turn, leads to the swollenness of the liver. A study found fatigue to be present in two thirds of patients with chronic Hepatitis C [131]. Another study reported that 53% of Hepatitis C patients suffer from fatigue. In 17% of these patients, fatigue was so severe that it led to activity impairment [132]. The only study reported in this review about Hepatitis C found the development of fatigue related to the ventral striatum [104], which mainly intercedes in reward cognition, reinforcement and motivation. HIV is a virus which attacks the immune system, which is the body’s natural defence against infections and diseases. Prevalence of fatigue in HIV has varying rates according to the stage of the disease, ranging from no fatigue in the early stage of the disease, to almost 80% in AIDS patients [8,133,134,135,136,137,138]. Just one study regarding HIV met the inclusion criteria of this review and hence, was assessed [105]. Here, basal ganglia was associated with fatigue. Patients who suffered from paralytic poliomyelitis can develop postpoliomyelitis syndrome (PPS) years or even decades later. PPS is a disorder where patients suffer from a generalized fatigue, generally reported as the most burdensome symptom of the disease [139]. Approximately 66% to 89% of PPS patients suffer from high levels of fatigue [140,141,142]. Two studies regarding PPS-related fatigue were assessed. However, just one of them found significant findings. Reticular formation, putamen and medial leminiscus were indicated as possible correlates of fatigue. These structures are thought to be involved in several functions, such as behavioural arousal and consciousness, regulation of movements and somatosensation from the skin and joints. Chronic fatigue syndrome is a complex disorder whose most common symptom is extreme fatigue. In the UK, the prevalence of CFS has been rated between 11% [143] and 15% [144] per 100,000 people. This disorder has been widely studied and it has been the most reported illness in this article. A total of 13 CFS studies related to fatigue were gathered. The majority of them found areas related to executive and cognitive functions, memory and perception.

The brain structures found to be related to fatigue in the studies compared were highly heterogeneous, not only between diseases, but also for different studies within the same diseases. The most common structures were the frontal lobe, parietal lobe, limbic system and basal ganglia. These structures are associated with attention, memory, planning, integration of sensory information and learning.

Longitudinal studies, as opposed to cross-sectional studies, allow tracking of the same subjects over time, removing confounders such as cultural differences, age, etc. Therefore, they offer more precise and reliable indicators. In addition, they can give information about accumulative processes. What is more, they may allow the prediction of future changes in fatigue by assessing baseline factors. In spite of this, just seven out of 26 studies were longitudinal [91,93,99,100,104,105,114], and six involved an interventional procedure. Within these six interventional studies, one was low quality and only employed MRS [105], one only recorded structural images and did not include a proper control group [104], and another only recorded structural images [93]. The fact that longitudinal designs allow follow-up of the patient during an intervention procedure, offering more precise indicators of the population changes, and their potential as prediction models, make them the best strategy to follow in the search of neural correlates of fatigue. Therefore, longitudinal neuroimaging studies following a fatigue treatment would be of inestimable worth in the study of this symptom.

sMRI, fMRI, DTI and FC studies identified significant neural correlates of fatigue, demonstrating that they are very useful techniques in the search for the processes responsible for fatigue within chronic illnesses. The integration of these techniques may be helpful in the search of biomarkers, rather than using them individually [145]. However, none of the studies in this review integrated all of them. sMRI, fMRI and DTI were integrated in [98] and sMRI, fMRI and FC in [106], both of them with no follow-up. Other studies have combined two of the techniques; sMRI and fMRI [96,108,111,112,113], sMRI and DTI [97,102,107], or sMRI and FC [40,72]. Integration of modalities is an attractive strategy to follow in the development of a comprehensive map of the pathophysiological brain networks of fatigue. This multimodal approach has demonstrated a more comprehensive understanding of brain changes in disorders such as amyotrophic lateral sclerosis [146], schizophrenia [147,148,149,150,151,152,153,154,155,156,157], bipolar disorder [158,159,160,161], characterization of tumours [162], traumatic brain injury [163], Parkinson’s disease [164], psychosis [165,166], Alzheimer’s disease [167,168] and mild cognitive impairment [169,170,171]. Therefore, it should be expected that the integration of techniques may help to elucidate further brain mechanisms of fatigue.

### 4.2. Limitations

This systematic review aimed to perform a comprehensive search of all studies employing MR techniques in the investigation of neural indicators of fatigue. Although a great number of references were gathered, it is still conceivable that other related papers have been overlooked. Furthermore, the inclusion criteria set a lower limit of studies with more than 10 subjects. This limit was based on fMRI which tend to have lower sample sizes than sMRI studies. This may mean that lower power, and therefore lower quality, sMRI studies may have been included than would otherwise be the case with a larger sample size threshold. We acknowledge the diversity in the way that fatigue was measured in the studies examined. Furthermore, studies employing fMRI make use of different tasks to induce fatigue. These facts increase the risk of bias and will inevitably contribute to a disparity in results. Finally, we acknowledge that the study of brain correlates by means of neuroimaging is insufficient to offer a comprehensive explanation of the mechanisms underlying fatigue. The integration of other types of biomarkers, such as biochemical ones, may provide further understanding of the phenomenon.

## 5. Conclusions

We have found that studies searching for neural indicators of fatigue within chronic diseases are of variable design and quality. Regarding the fatigue indicators, we found that there are not well-defined neural correlates of fatigue in any chronic diseases so far. Different designs and methodologies for the same illness offered different results. From this, we conclude that there are no common correlates of fatigue across chronic diseases. According to the employed design, it seems that the best strategy in the search of neural correlates of fatigue would be to integrate different neuroimaging techniques in a longitudinal study, with a fatigue alleviating intervention. Such an approach could be a great asset to unravel the neural mechanisms of this burdensome and neglected symptom.

## Figures and Tables

**Table 1:** Characteristics of included studies.

Ref.	Disease	User Group			Control Group			Design	Follow-Up	Task	Fatigue Assessment	Modality	Statistical Method
		*n*	Male/ Female	Age Mean (std)	*n*	Male/Female	Age Mean (std)						
[93]	AS	129 TNF-treated	95/34	43.6 (11.4)	NA	NA	NA	Cross-sectional	NA	NA	FSS	NA	Pearson test, Student *t* test, paired *t* test, Wilcoxon signed-rank test, Spearman’s rank order correlation, forward stepwise selection in multivariate GLM
		14	11/3	37.6 (11.9)	14	11/3	37.2 (10.2)	Longitu-dinal	At baseline and 4 months after the start of TNF treatment	NA	FSS	sMRI	
[97]	AS	20	15/5	34.8 (11.9)	20	15/5	34.9 (9.6)	Cross-sectional	NA	NA	FSS	sMRI, DTI	MonteCarlo simulations, Spearman’s correlation, multiple stepwise regression analysis
[98]	Cancer	32 and 33 BC scheduled and not indicated to receive ChT	0/32 0/33	50.2 (9.2) (Pre-ChT+) 52.4 (7.3) (Pre-ChT-)	38	0/38	50.1 (8.7)	Cross-sectional	NA	ToL, Paired Associates Memory Task	CFS	sMRI, FLAIR, 1H-MRS, PRESS, DTI, fMRI	ANOVA, Chi-squared test, z-scores, Mahalanobis Distance, logistic regression, variance-covariance matrix
[99]	Cancer	28 and 37 treated with and without ChT	0/28 0/37	50.0 (10) (ChT) 53.0 (9) (No ChT)	32	0/32	50.0 (9)	Longitu-dinal	1 month post-ChT (aprox. 5 months between scans)	VWMT	FACIT-F	fMRI	Multiple linear regression analysis, *t* tests, ANOVA, Pearson correlation
[100]	Cancer	20 fatigued cancer survivors	10/10	47.9 (10.1)	20 non fatigue cancer survivors	10/10	48.9 (9.7)	Cross-sectional	NA	NA	CIS-fatigue	NA	Shapiro-Wilk test, Chi square tests, independent samples *t* tests, Mann-Whitney U tests, Wilcoxon matched-pairs tests
		25 fatigued cancer survivors (selected for intervention)	14/11	48.8 (9.4)	14 fatigued cancer survivors (selected for waiting list)	5/9	50.6 (10.9)	Longitu-dinal	At baseline and 6 months later	CBT (for the user group)		sMRI, 1H-MRS	
[101]	PBC	14 PBC (stage I–II disease) 4 PBC (stage III–IV)	0/14 0/4	60.0 (–) (41–76) ^a^ 48.0 (–) (39–59) ^a^	11 HC	0/11	47 (–) (38–65) ^a^	Cross-sectional	NA	NA	FIS	sMRI, 1H- MRS, MTR	Shapiro-Wilk test, Student’s *t* test, Mann-Whitney U test, Pearson’s correlation
[96]	GPA	12 fatigued 16 non fatigued	6/6 6/8	58.5 (15.9) (fatigued) 51.6 (13.8) (non fatigued)	13 general popula-tion with idio-pathic fatigue	7/6	52.2 (10.5)	Cross-sectional	NA	PASAT	CFS	sMRI, fMRI	Fisher’s exact tests, *t* tests, Mann-Whitney tests, MonteCarlo simulations
[102]	GPA	14 GPA with chronic fatigue	6/8	58.6 (15.1)	14 GPA without fatigue	6/8	51.6 (13.8)	Cross-sectional	NA	NA	CFS	sMRI, DTI, FLAIR	Mann-Whitney tests, *t* tests, *x*^2^ tests
[103]	Gulf War Illness	31	11/9	45.9 (–) (43.2–48.4) ^a^	20	25.6	45.6 (–) (41.2–50.5) ^a^	Cross-sectional	NA	NA	Ordinal fatigue rating, CFS, MFI, SF-36	DTI	Student’s *t* test, Fisher’s exac*t* tests, *p* values, Bonferroni corrections, ROC, Pearson’s function, Spearman’s function, stepwise multivariate linear regression analysis
[104]	Hepatitis C	23 initiation IFN-α treatment (19 completed both MRI scans, and 20 both blood samples)	17/6	48.8 (10.9)	NA	NA	NA	Longitu-dinal	qMT and blood sampling at baseline and 4 h after IFN-α injection. Behavioural and psychological assessments at both scanning sessions and at treatment weeks 4, 8, 12 and 24	NA	VAS-f	sMRI, qMT	ANOVA, paired sample *t*-tests, regression analysis, Mauchly’s sphericity test, Levenberg-Marquardt nonlinear least squares, FEW
[105]	HIV	82 fatigued HIV patients	71/11	44.0 (41–50) ^b^	46 non- fatigued HIV patients	41/5	48.0 (43–54) ^b^	Longitu-dinal	At baseline, and weeks 12 and 24 (Just 62 of the 128 patients underwent 1H-MRS)	NA	FSS	MRS	Kuskal-Wallis tests, Score tests, GEE models
[95]	PPS	42 PPS 49 MS	15/27 (PPS) 17/32 (MS)	60.86 (7.65) (PPS) 46.18 (9.4) (MS)	27	11/16	46.96 (14.58)	Cross-sectional	NA	NA	FSS	sMRI	Multivariate linear regression, Spearman correlation, unpaired *t*-test
[92]	PPS	22	–	–	NA	NA	NA	Cross-sectional	NA	NA	Postpolio fatigue questionnaire	sMRI	Produce moment correlations, linear regression, independent *t* tests
[39]	CFS	17 ME/CFS	0/17	49.82 (11.78)	17 HC	0/17	48.88 (12)	Cross-sectional	NA	NA	FFQ, VAS	sMRI, pCASL FC	Spearman’s rho
[40]	CFS	19 ME/CFS	0/19	52.33 (10.63)	17 HC	0/17	48.75 (11.75)	Cross-sectional	NA	NA	MFI	sMRI, ASL FC, BOLD FC	*t*-tests, ICA, Pearson
[106]	CFS	18	0/18	43.9 (4.8)	18 HC	0/18	45.9 (3.2)	Cross-sectional	NA	6 min passive-viewing block scan	CFS	sMRI, fMRI, FC	Fisher, independent *t*-tests, ANOVA
[107]	CFS	15	7/8	46.5 (13.2)	14	6/8	46.6 (14.6)	Cross-sectional	NA	NA	MFI-20	sMRI, DTI, ASL	Pearson correlation, *t* tests, ROC curve
[108]	CFS	18	2/16	44.2 (11.1)	41 HC	8/33	47.2 (9.2)	Cross-sectional	NA	Gambling	MFI-20, SF-36	sMRI, fMRI	*t*-test, Chi-square test, Fisher exac*t* test, Welch *t*-test, MANCOVA, Bravais-Pearson correlation
[109]	CFS	25	6/19	31.7 (8.8)	25 HC	6/19	33.7 (10.3)	Cross-sectional	NA	NA	CFS fatigue duration	sMRI	Regressions, Bonferroni corrected *p* values
[110]	CFS	12	4/8	33.75 (7.64)	11 HC	4/7	34.36 (6.77)	Cross-sectional	NA	Fatigue and anxiety provocation task	CFS, PF-SF36	sMRI, fMRI	Student’s *t* tests, *x*^2^, ANOVA
[91]	CFS	22	0/22	36.6 (2.5)	22 HC	0/22	37.1 (2.2)	Longitu-dinal	Before and after CBT (6–9 months)	NA	Physical assessment (actometer), perceived fatigue severity (checklist individual strength)	sMRI	Tailed multivariate linear regression analysis, *t*-tests, family-wise error correction, Spearman’s correlation, Mahalanobis distance to check for multivariate outliers
[111]	CFS	17	7/10	35.53 (6.17)	12 HC	4/8	33.5 (7.12)	Cross-sectional	NA	n-Back task	PF-SF36, CFS	sMRI, fMRI	Student *t* test, *x*^2^, Mann-Whitney U tests, Wilcoxon test
[112]	CFS	6 CFS with verbal working memory difficulties according to PASAT	0/6	38.17 (9)	7	3/4	30.71 (9.6)	Cross-sectional	(scan) Baseline → task1 → task2 → task1 → task2	Auditory monitoring test,	Neuropsychological testing	sMRI,	Student *t* test, analysis of covariance
		19 CFS without verbal memory difficulties	3/16	37.53 (8)	15	5/10	30.80 (7.5)	Cross-sectional	(scan) Baseline → task1 → task2 → task1 → task2 (STAI) before and after scanner	mPASAT, BDI, STAI	Neropsychological testing MFI-20	fMRI	
[94]	CFS	16	10/6	34.0 (-)	49 HC	27/22	34.44 (–)	Cross-sectional	NA	NA	Self-reported ratings based on daily activities	sMRI	Permutation tests, Spearman’s rank correlation coefficient
[113]	CFS	16	0/16	28.4 (6)	16 HC	0/16	24.9 (6.4)	Cross-sectional	NA	Motor and visual imagery task	CIS-R, mean actometer score	sMRI, fMRI	GLM, regressions MANOVA, ANCOVA
[114]	CFS	15 without depression 11 with depression	7/8 1/10	28.4 (–) (25.5–31.3) ^a^ 31.3 (–) (27.7–34.8) ^a^	18 HC	3/15	32.9 (–) (29.3–36.5) ^a^	Longitu-dinal	Cognitive testing at baseline and 3–6 months later (just for 14 subjects)	NA	fatigue questionnaire	sMRI	ANOVA, multiple linear regression analysis

ANOVA: Analysis of Variance; AS: Ankylosing Spondylitis; ASL: Arterial Spin Labelling; BC: breast cancer; BOLD: Blood Oxygen Level Dependent; CBT: Cognitive Behaviour Therapy; CFS: Chronic Fatigue Syndrome or Chalder Fatigue Scale; ChT: chemotherapy; CIS: Checklist Individual Strength; DTI: Diffusion Tensor Imaging; FACIT-F: Functional Assessment of Chronic Illness Therapy—fatigue; FC: functional connectivity; FEW: Family Wise Error; FFQ: Florida Fatigue Questionnaire; FIS: Fatigue Impact Scale; FLAIR: Fluid-attenuated inversion recovery; fMRI: functional Magnetic Resonance Imaging; FSS: Fatigue Severity Scale; GEE: Generalized Estimating Equations; GLM: General Linear Modelling; GPA: Granulomatosis with Poliangiitis; HC: Healthy Control; HIV: Human Immunodeficiency Virus; ICA: Independent Component Analysis; IFN-α: Interferon—α; MANCOVA: Multivariate Analysis of Variance; MFI: Multidimensional Fatigue Inventory; MRS: Magnetic Resonance Spectroscopy; MS: Multiple Sclerosis; MTR: Magnetization Transfer Ratio; NA: Not applicable; PASAT: Paced Auditory Serial Attention Task; PBC: Primary Biliary Cirrhosis; pCASL: pseudo-Continuous Arterial Spin Labelling; PF-SF36: Physical Functioning scale from the 36-item Short Form Health Survey; PPS: Postpoliomyelitis syndrome; PRESS: Point Resolved Spectroscopy; qMT: quantitative Magnetization Transfer; ROC: Receiver Operating Characteristic; SF-36: Short Form 36; sMRI: structural Magnetic Resonance Imaging; STAI: State and Trait Anxiety Inventory; TNF: Tumor Necrosis Factor; ToL: Tower of London; VAS-f: Visual Analogue Scale—fatigue; VWMT: Verbal Working Memory Task. ^a^ Mean plus range; ^b^ Median plus IQR.

**Table 2:** Quality assessment of included studies.

Reference	Year	Pathology	Design	Scoring Criteria for Quality Assessment										Score
				1	2	3	4	5	6	7	8	9	10	(%)
[93]	2015	AS	Cross-sectional	Y	Y	Y	Y	Y	Y	N	Y	Y	N	100
			Longitudinal							Y				
[97]	2014	AS	Cross-sectional	Y	Y	Y	Y	Y	Y	Y	Y	Y	Y	100
[98]	2015	Cancer	Cross-sectional	Y	Y	Y	Y	Y	Y	Y	Y	Y	Y	100
[99]	2014	Cancer	Longitudinal	Y	Y	Y	Y	Y	N	Y	Y	Y	Y	90
[100]	2013	Cancer	Cross-sectional	Y	Y	Y	Y	Y	Y	Y	Y	Y	Y	100
			Longitudinal											
[101]	2004	PBC	Cross-sectional	Y	Y	Y	Y	Y	Y	Y	Y	Y	Y	100
[96]	2014	GPA	Cross-sectional	Y	Y	Y	Y	Y	Y	Y	Y	Y	Y	100
[102]	2013	GPA	Cross-sectional	Y	Y	Y	Y	Y	N	Y	Y	Y	Y	90
[103]	2013	Gulf War Illness	Cross-sectional	Y	Y	Y	Y	Y	Y	Y	Y	Y	Y	100
[104]	2016	Hepatitis C	Longitudinal	Y	Y	Y	Y	Y	Y	N	Y	Y	Y	90
[105]	2010	HIV	Longitudinal	Y	N	N	Y	Y	N	Y	N	Y	N	50
[95]	2014	PPS	Cross-sectional	Y	Y	Y	Y	Y	Y	Y	Y	Y	Y	100
[92]	1994	PPS	Cross-sectional	Y	Y	Y	N	Y	Y	N	Y	Y	Y	80
[39]	2016	CFS	Cross-sectional	Y	Y	Y	Y	Y	Y	Y	Y	Y	Y	100
[40]	2016	CFS	Cross-sectional	Y	Y	Y	Y	Y	Y	Y	Y	Y	Y	100
[106]	2015	CFS	Cross-sectional	Y	Y	Y	Y	Y	Y	Y	Y	Y	Y	100
[107]	2015	CFS	Cross-sectional	Y	Y	Y	Y	Y	Y	Y	Y	Y	Y	100
[108]	2014	CFS	Cross-sectional	Y	Y	Y	Y	Y	Y	Y	Y	Y	Y	100
[109]	2011	CFS	Cross-sectional	Y	Y	Y	Y	Y	Y	Y	Y	Y	N	90
[110]	2008	CFS	Cross-sectional	Y	Y	Y	Y	Y	Y	Y	Y	Y	Y	100
[91]	2008	CFS	Longitudinal	Y	Y	Y	Y	Y	Y	Y	Y	Y	Y	100
[111]	2006	CFS	Cross-sectional	Y	Y	Y	Y	Y	Y	Y	Y	Y	Y	100
[112]	2005	CFS	Cross-sectional	Y	Y	Y	Y	Y	Y	Y	Y	Y	Y	100
[94]	2004	CFS	Cross-sectional	Y	Y	N	Y	Y	Y	Y	Y	Y	N	80
[113]	2004	CFS	Cross-sectional	Y	Y	Y	Y	Y	Y	Y	Y	Y	Y	100
[114]	1995	CFS	Longitudinal	Y	Y	Y	Y	Y	Y	Y	Y	Y	Y	100

Assessment criteria questions: (1) Does the study have a clearly defined research objective? (2) Does the study adequately describe the inclusion criteria? (3) Does the study adequately describe the exclusion criteria? (4) Does the study report on the population parameters/demographics? (5) Does the study report details on assessment of pain? (6) Does the study provide details of imaging protocol? (7) Does the study provide a proper control group? (8) Does the study apply proper statistical analysis? Correction for multiple comparisons? (9) Does the study adequately report on the strength of the results (e.g., ways of calculating effect sizes, reporting of confidence intervals/standard deviation)? (10) Do the authors report on the limitations of their study? Y = yes, N = no, Y/N = applies partially; AS: Ankylosing Spondylitis; CFS: Chronic Fatigue Syndrome; GPA; Granulomatosis with Poliangiitis; HIV: Human Immunodeficiency Virus; PBC: Primary Biliary Cirrhosis; PPS: Pospoliomyelitis Syndrome.

**Table 3:** Results of brain indicators of fatigue for each study.

Reference	Pathology	Summary of Key Neuroimaging Findings Related to Fatigue	Quality Score (/10)
[93]	AS	Negative correlation between fatigue reduction after anti TNF-α therapy and cortical thickness of the insula, primary sensory cortex/inferior parietal sulcus and superior temporal polysensory areas.	100
[97]	AS	Negative correlation between fatigue scores and amount of GM in areas of the dorsal and ventral attention networks, the somatosensory cortices, and the caudate nucleus. Positive correlation between fatigue scores and GM within the executive control network and putamen.	100
[98]	Cancer	Positive correlation between fatigue and ToL task BOLD activation across groups in the dorsomedial prefrontal cortex.	100
[99]	Cancer	Prediction of post-treatment fatigue severity by pre-treatment spatial variance in executive network activation.	90
[100]	Cancer	No significant findings.	100
[101]	PBC	Positive correlation between fatigue score and blood manganese and copper concentrations. Significant reduction in globus pallidus/WM and globus pallidus/PU MTR indices in the high fatigue group compared with the low fatigue group, in stage I–II patients.	100
[96]	GPA	↑ activation in the right thalamus, left paracentral lobule, left medial frontal gyrus and right medial globus pallidus among GPA cases compared with GPA controls.	100
[102]	GPA	↑ structural integrity in fornix and cingulum among GPA cases.	90
[103]	Gulf War Illness	Positive correlation of fatigue, pain, and ↑ axial diffusivity with the right inferior fronto-occipital fasciculus.	100
[104]	Hepatitis C	Correlations bilaterally between shifts in kf and T2f within the ventral striatum and the subsequent development of fatigue.	90
[105]	HIV	↓ levels of the cellular energy marker total creatine in the basal ganglia within fatigued participants.	50
[95]	PPS	No significant findings.	100
[92]	PPS	Small discrete or multiple punctate areas of hyperintense signal (HS) in the reticular formation, putamen, medial leminiscus or WM tracts imaged in 55% of the subjects reporting ↑ fatigue and none in those reporting ↓ fatigue.	80
[39]	CFS	Negative correlation between fatigue ratings and connectivity between left parahippocampal gyrus connectivity and left postcentral gyrus and left supra-marginal gyrus. Positive correlation between fatigue and connectivity of anterior cingulate cortex withthe posterior cingulate cortex, left thalamus, and left hippocampus.	100
[40]	CFS	Negative correlation between fatigue and fC between salience network and posterior cingulate cortex. Negative correlation between fatigue and fC between resting state network and anterior midcingulate cortex.	100
[106]	CFS	Positive correlation between fatigue and connectivity between posterior cingulate cortex and dorsal anterior cingulate cortex.	100
[107]	CFS	No significant findings.	100
[108]	CFS	Negative correlation between fatigue and activation in the right globus pallidus.	100
[109]	CFS	Negative correlation between fatigue duration and WM volume in the midbrain.	90
[110]	CFS	During provocation of fatigue, ↑ activation in the occipito-parietal cortex, posterior cingulate gyrus and parahippocampal gyrus, and ↓ activation in dorsolateral and dorsomedial prefrontal cortices in CFS compared to controls.	100
[91]	CFS	Significant ↑ in GM volume, localized in the lateral prefrontal cortex in CFS cases, with CBT.	100
[111]	CFS	During 1-back condition, ↑ activation in medial prefrontal regions, including the anterior cingulate gyrus, in CFS cases compared to control subjects. On more challenging conditions, ↓ activation in dorsolateral prefrontal and parietal cortices in CFS cases. On the 2- and 3-back conditions, significant activation of a large cluster in the right inferior/medial temporal cortex in CFS cases.	100
[112]	CFS	Positive correlation between fatigue and BOLD signal change in the left superior parietal region, bilateral supplemental and premotor regions.	100
[94]	CFS	Negative correlation between fatigue and right dorsolateral prefrontal-cortex.	80
[113]	CFS	No significant findings	100
[114]	CFS	White-matter lesions in a minority from all groups.	100

AS: Ankylosing Spondylitis; BOLD: Blood Oxygen Level Dependent; CBT: Cognitive Behavioural Therapy; CFS: Chronic Fatigue Syndrome; FC: functional connectivity; GM: Grey Matter; GPA; Granulomatosis with Poliangiitis; HIV: Human Immunodeficiency Virus; MFI: Multidimensional Fatigue Inventory; MTR: Magnetization Transfer Ratio; PBC: Primary Biliary Cirrhosis; PPS: Pospoliomyelitis Syndrome; TOL: Tower of London; WM: White Matter.

## Abbreviations

ALS	Amyotrophic Lateral Sclerosis
AS	Ankylosing Spondylitis
BOLD	Blood Oxygen Level Dependent
CFS	Chronic Fatigue Syndrome
CNS	Central Nervous System
CT	Computed Tomography
DTI	Diffusion Tensor Imaging
FC	Functional Connectivity
FM	Fibromyalgia
fMRI	Functional Magnetic Resonance Imaging
GM	Grey Matter
GPA	Granulomatosis with Polyangiitis
HIV	Human Immunodeficiency Virus
MR	Magnetic Resonance
MRI	Magnetic Resonance Imaging
MRS	Magnetic Resonance Spectroscopy
MS	Multiple Sclerosis
PASAT	Paced Auditory Serial Attention Test
PET	Positron Emission Tomography
PRISMA	Preferred Reporting Items for Systematic Reviews and Meta-Analyses
qMT	Quantitative Magnetization Transfer
RA	Rheumatoid Arthritis
SLE	Systemic Lupus Erythematosus
sMRI	Structural Magnetic Resonance Imaging
SR	Systematic Review
VBM	Voxel Based Morphometry
WM	White Matter
